# Readability of Online Patient Education Materials for Interventional Pain Procedures

**DOI:** 10.7759/cureus.10684

**Published:** 2020-09-27

**Authors:** Dilip Kamath, Sean Mcintyre, Evan Peskin, Scott Stratman, Nitin Agarwal, Preetha D Kamath, Raghav Gupta, Ruben Schwartz, Alaa Abd-Elsayed, Ivan Urits, Omar Viswanath, Alan D Kaye, Danielle B Horn

**Affiliations:** 1 Anesthesiology, Jackson Memorial Hospital, Miami, USA; 2 Anesthesiology, Herbert Wertheim College of Medicine, Florida International University, Miami, USA; 3 Anesthesiology, University of Miami, Miami, USA; 4 Neurosurgery, University of Pittsburgh, Pittsburgh, USA; 5 Dermatology, University of Miami Miller School of Medicine, Miami, USA; 6 Neurosurgery, Rutgers University, Newark, USA; 7 Anesthesiology, Mount Sinai Medical Center of Florida, Miami, USA; 8 Anesthesiology and Pain Management, University of Wisconsin, Madison, USA; 9 Anesthesia, Critical Care and Pain Medicine, Beth Israel Deaconess Medical Center, Harvard Medical School, Boston, USA; 10 Pain Management, Valley Pain Consultants - Envision Physician Services, Phoenix, USA; 11 Anesthesiology, Louisiana State University Health Sciences Center, Shreveport, USA; 12 Pain Management, Jackson Memorial Hospital, Miami, USA

**Keywords:** readability

## Abstract

Background

The internet has had an enormous influence on the field of medicine. In this regard, Statista, a market and consumer data company, estimated in 2019 that more than half the world’s population (>four billion people) were active internet users. Accessing the World Wide Web has become the second nature for most. In the medical field, many patients look to the internet for information regarding certain procedures. The purpose of this study, therefore, was to assess the readability level of more than 492 online sources with information on a wide array of interventional pain procedures.

Objective

The aim is to determine the readability of online patient educational materials for interventional pain procedures.

Study design

This is a retrospective review.

Methods

We downloaded and reformatted to plain text 492 internet-based patient educational materials for 17 different interventional pain procedures. Plain text was processed using Readability Studio (Oleander Software Ltd., Vandalia, Ohio, USA), which employs 10 quantitative readability scales that are widely used and accepted in the medical literature.

Results

The software determined the average reading level (or grade level) of the 492 online sources we examined to be 12.1, with a range of 10.9 to 13.

Limitations

Google is not the only online search engine patients utilize for information, and the top links for each search could change over time. Also, some patients prefer videos rather than text to learn about their disease and treatment options. Finally, depending on their provider, the links that patients are directed to may be significantly more or less readable.

Conclusions

The average American adult reads at the eighth grade level. The National Institutes of Health and the American Medical Association have recommended education materials be made available at the third to seventh grade level. Our analysis shows patient educational materials found online for interventional pain procedures to be overly technical, with an average reading level (or grade level) of 12.1.

## Introduction

The internet has had an enormous influence on the field of medicine. In this regard, Statista, a market and consumer data company, estimated in 2019 that more than half the world’s population (>four billion people) were active internet users [1]. It is no surprise that patients are increasingly accessing available information to better understand and participate in their healthcare with the expansion of health-related educational materials for patients [2]. According to a report published by Rainie and Fox, 70% of responders reported that Web information influenced their decision about how to treat a condition or illness, and 81% found the information they desired through an Internet search rather their healthcare provider [3]. However, improved access to information does not guarantee patients a better understanding of their diseases or treatment options. Not only can they be exposed to misinformation, but comprehensible and readable educational materials are also lacking [4-6]. Because the average American adult reads at the eighth grade level, the United States National Institutes of Health (NIH) and the American Medical Association (AMA) have recommended education materials to be made available at the third to seventh grade level. The purpose of this study, therefore, was to assess the readability level of more than 492 online sources with information on a wide array of interventional pain procedures.

## Materials and methods

All data in the present investigation were publicly available through internet search using the Google search engine. As such, no Institutional Review Board approval was required. From June to September 2019, we downloaded 492 internet-based patient educational materials for 17 different interventional pain procedures. The search was executed using the following phrases: “spinal cord stimulator”, “intrathecal pump”, “epidural steroid injection”, facet joint injection”, “genicular nerve block”, “medial branch nerve block”, “medial branch nerve radiofrequency ablation”, “lateral branch nerve radiofrequency ablation”, “suprascapular nerve radiofrequency ablation”, “intraarticular knee injection”, “intraarticular hip injection”, “glenohumeral joint injection”, “lumbar sympathetic block”, “piriformis injection”, “stellate ganglion block”, “intercostal nerve block”, and “occipital nerve block”. The top links for each search term, up to a limit of 30, were accessed, and patient-specific information with regard to each procedure was downloaded. The text was then reformatted to plain text using word processing software (Microsoft Word). Hyperlinks, figures, legends, and text unrelated to patient education were discarded. The final reformatted text was processed using Readability Studio (Oleander Software Ltd., Vandalia, Ohio, USA), which employs 10 quantitative readability scales that are widely used and accepted in the medical literature.

## Results

The Readability Studio composite of eight readability assessments determined the average reading level (or grade level) of the 492 online sources we examined, which were patient-specific educational materials on interventional pain procedures, to be 12.1, with a range of 10.9 to 13. The Flesch Reading Ease assessment, which is commonly used in health literacy literature, found the text to be “difficult” to read. Table 1 summarizes the formulas for readability assessment algorithms used by each assessment strategy. Table 2 demonstrates the readability assessment scores determined by each assessment database.

**Table 1:** Readability Assessment Algorithms A, mean number of words per sentence; B, mean number of words with three or more syllables; C, mean number of sentences per 100 words; D, number of complex words; E, mean number of letters per 100 words; F, mean number of sentences; G, number of single-syllable words in a 150-word sample; H, mean number of words; I, percentage of unfamiliar words within text; J, number of sentences; K, number of “easy” words; L, number of polysyllabic words; M, average word length; N, average familiar words

Readability Assessment Scale	Formula
Flesch reading ease	206.835 - [1.015 x K - (84.6 x A)]
Coleman-Liau index	(0.0588 x E) - (0.296 x C) - 15.8
Degrees of reading power (grade equivalent)	0.886593 - (M x 0.03640) + (N x 0.161911) - (A x 0.21401) - (A x 0.000577) - (A x 0.000005)
FORCAST readability formula	20 - (G/10)
Fry readability formula	100-word passage from selection selected; number of sentences in passage estimated; number of syllables in each passage determined; chart used to identify readability based on calculated metrics
Gunning fog index	0.4 x [A/F + (100 x B/A)]
New Dale-Chall formula	(0.0496 x H) + (0.1579 x I) + 3.6365
Raygor readability estimate	100-word passage from selection selected; number of sentences in passage estimated; number of 6 or more letter words determined; chart used to identify readability based on calculated metrics
SMOG index	1.043 x sqroot [L x (30/F)] + 3.1291

**Table 2:** Readability Assessment Scale Scores *The Flesch reading ease is scored on a 0 to 100 scale, with lower scores indicating more difficult text and higher scores indicating more readable text (0-29, very difficult; 30-49, difficult; 50-59, fairly difficult; 60-69, standard; 70-79, fairly easy; 80-89, easy; 90-100, very easy).

Readability Assessment Scale	Score, Mean, Grade Level
Flesch reading ease	49*
Coleman-Liau index	11.9
Degrees of reading power (grade equivalent)	12.5
FORCAST readability formula	10.9
Fry readability formula	13
Gunning fog index	12.4
New Dale-Chall formula	12
Raygor readability estimate	11
SMOG index	13
Overall	12.1

## Discussion

Our analysis shows that the information patients may obtain online regarding interventional pain procedures is overly technical according to NIH and AMA standards. Readability is an important component of health literacy, and studies have demonstrated low health literacy to be associated with poorer health outcomes and poorer use of health services [5]. This is particularly important with respect to persons from lower socioeconomic classes, who are already at a disadvantage when receiving appropriate healthcare, and immigrants who may not speak perfect English.

When meeting a patient for the first time, it is important to attempt to understand what they may think regarding a specific treatment initially. If it is evident that the patient does not fully comprehend any of the available and subsequently recommended treatment options, an opportunity exists to clarify this confusion. By providing them with the knowledge they require to make the best decision for themselves regarding their health, this will likely improve trust in the practitioner, increase patient satisfaction, and ensure patient autonomy. Additionally, there is clearly a need to improve patient access to appropriate, accurate third to seventh grade level patient-specific information regarding these interventional pain procedures. If created, these documents could be distributed to the patients prior to their proposed procedure appointment.

## Conclusions

The average American adult reads at the eighth grade level. The NIH and AMA have recommended education materials be made available at the third to seventh grade level. Our analysis shows patient educational materials found online for interventional pain procedures to be overly technical, with an average reading level (or grade level) of 12.1.
